# Anlotinib combined with Sintilimab is win-win cooperation for primary squamous cell carcinoma of the thyroid: A case report and literature review

**DOI:** 10.3389/fonc.2023.976415

**Published:** 2023-03-16

**Authors:** Zichang Liu, Maosheng Yu, Feng Zhao, Chenfang Zhu

**Affiliations:** Department of General Surgery, Shanghai Ninth People’s Hospital, Shanghai Jiao Tong University School of Medicine, Shanghai, China

**Keywords:** primary squamous cell carcinoma of the thyroid, tyrosine kinase inhibitors, immune checkpoint inhibitors, Anlotinib, Sintilimab, immune-related adverse reactions, autoimmune liver disease

## Abstract

**Background:**

Primary squamous cell carcinoma of the thyroid (PSCCT) is a rare malignant tumor. The incidence rate of PSCCT is less than 1%. However, the diagnosis and treatment of PSCCT are limited. Surgical resection is considered to be one of the few effective intervention methods. In this article, we reported a case of taking tyrosine kinase inhibitors (TKIs) combined with immune checkpoint inhibitors (ICIs) for PSCCT.

**Case summary:**

An 80-year-old male was admitted to our hospital with dyspnea, cough, wheezing, and hoarseness for a giant thyroid mass. He underwent bronchoscopy and tracheal stent implantation to alleviate the respiratory obstruction. Then he accepted right partial thyroid and right lymph node biopsy. Postoperative pathology revealed squamous cell carcinoma. Subsequently, he underwent an endoscopy to exclude upper gastrointestinal squamous cell carcinoma. Finally, he was diagnosed with PSCCT. The patient was tentatively treated with a combination of Anlotinib and Sintilimab. After two courses, the tumor volume significantly reduced in MRI images and shrank further after five courses of combined treatment. Unfortunately, the patient died of fulminant liver failure and autoimmune liver disease after 5-month-treatment.

**Conclusion:**

TKIs combined with ICIs may be an effective and novel way for PSCCT treatment, but immune-related complications, especially liver damage, should be cared.

## 1 Introduction

PSCCT is a rare disease, which is less than 1% of all thyroid neoplasms (1). Due to the rapid growth of the tumor, invasion of surrounding structures is usually observed, which could induce a poor prognosis. The median survival time is generally shorter than one year (2, 3). Diagnosis is difficult because squamous metaplasia is common in other primary thyroid cancers, and squamous cell carcinoma may have metastasized from elsewhere (4). Thus, bronchoscopy and gastrointestinal endoscopy are necessary for excluding other primary squamous cell carcinomas from the upper respiratory and digestive tract (1, 4). PSCCT has a poor response to radiotherapy and is resistant to chemotherapy. Some articles reported surgery appeared to be one of the few methods that can reduce tumor burden and local invasion, prolonging survival (5–7).

In this case, we treated PSCCT with Anlotinib plus Sintilimab. We reported the symptoms, histopathological findings, and diagnostic procedures. The combination of medicines rapidly reduced the tumor volume. We also reviewed the literature to evaluate the conventional treatment of PSCCT and the progression of TKIs in combination with ICIs in other solid tumors.

## 2 Case presentation

**Figure 1:**
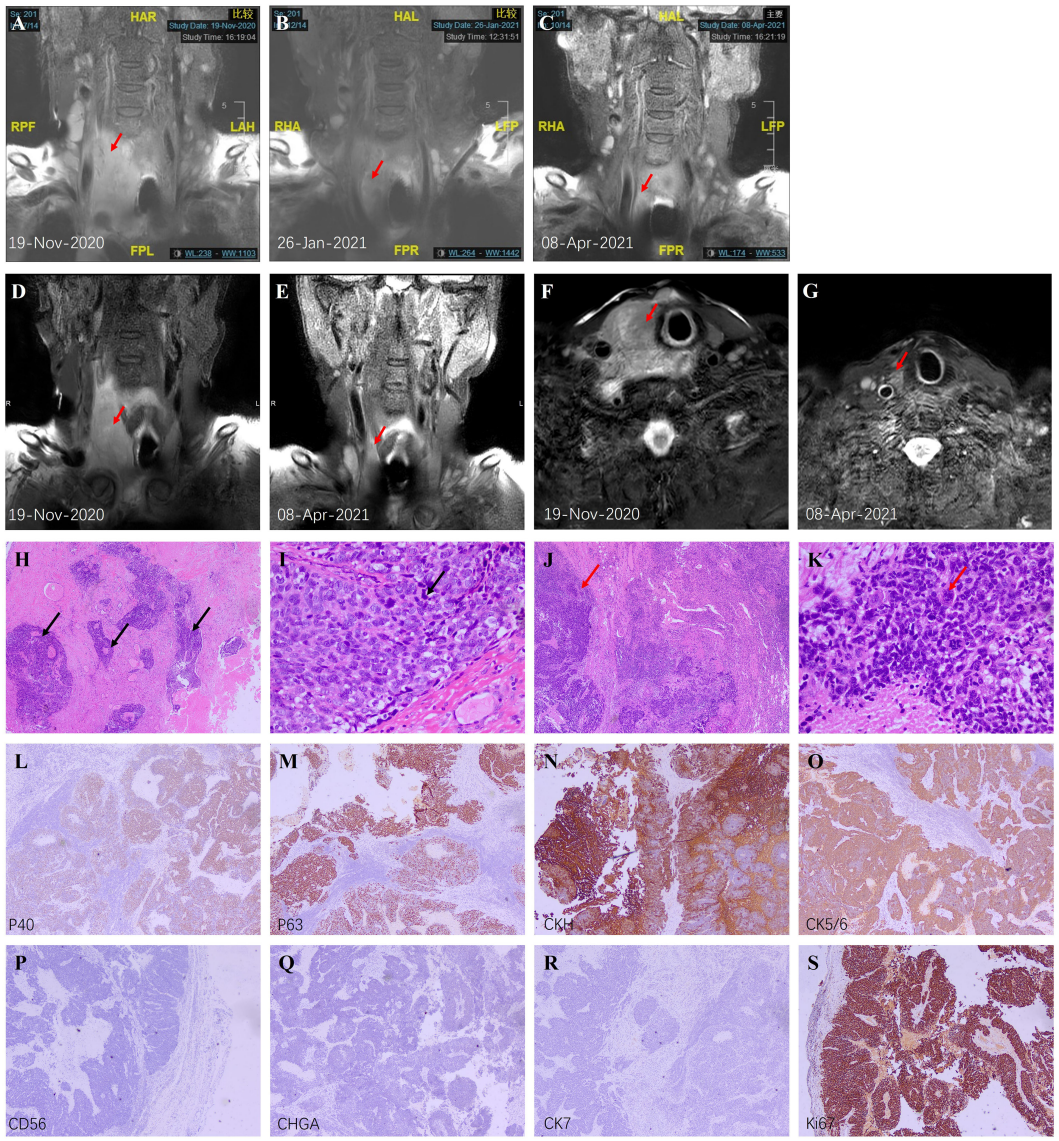
Imaging and pathological findings. **(A–C)** Coronal T2-weighted MRI scans before treatment, after 2 courses, and after 5 courses; **(D, E)** Coronal high-resolution modified Dixon T1-weighted MRI scans of thyroid before and after 5 cycles; **(F, G)** Axial high-resolution modified Dixon T2-weighted MRI scans of thyroid before and after 5 cycles. The red arrows indicate the thyroid nodule. **(H)** Low magnification of PSCCT (H&E, ×40) showed scattered islands of squamous cells (black arrows) with few typical follicular structures. **(I)** High magnification of PSCCT (H&E, ×400) showed poorly differentiated tumor cells and typical nuclear heterogeneous cells (black arrows). **(J)** Low magnification of right lymph node metastasis (H&E, ×40) showed diffuse proliferation of tumor tissue (red arrow). **(K)** High magnification of right lymph node metastasis (H&E, ×400) showed epithelioid appearing neoplastic cells lacking differentiation, and abnormal mitotic figures (red arrow) could be seen. **(L–S)** Immunohistochemistry of the right lymph node metastasis (×40). P40 **(L)**, P63 **(M)**, CKH **(N)**, and CK5/6 **(O)** were positive, indicating the source of squamous cells. CD56 **(P)** and CHGA **(Q)** are markers of neuroendocrine cells, while CK7 **(R)** is a marker of adenocarcinoma. Negative results suggested the low possibility of these two sources. **(S)** Ki67 index was over 95% suggesting vigorous proliferation excluding simple squamous metaplasia.

An 80-year-old male was admitted to our hospital for shortness of breath, cough, asthma, and hoarseness on November 19, 2020. Ultrasound showed a mass in the right lobe of the thyroid, about 38*51*78mm in size, partially involving the isthmus. Further MRI scans revealed a large right thyroid mass pressing on the airway (**Figures 1A, D, F**). Because of severe stenosis of the main trachea leading to chest tightness, palpitation, cough, and asthma, the patient was placed with a tracheal stent. Preoperative bronchoscopy showed that the vocal cords were fixed and compressed, and no new organisms were found in the airway wall. Owning to the failure of histopathological diagnosis by core needle biopsy, the patient underwent right partial thyroid and right neck lymph node surgical biopsy on November 25, 2020. Postoperative pathological examination revealed poorly differentiated squamous cell carcinoma. Scattered squamous cell islands and nuclear heterogeneous cells were seen. The right lymph node revealed a small round epithelial malignancy with low differentiation, and pathological mitotic figures could be seen (**Figures 1H–K**). Immunohistochemical staining was positive for CK5/6, CKH, P63, P40, CD5, CD117, and the Ki67 index was over 95%, while CK7, S100, CD56, SYN, NSE, and CHGA were negative. The results were consistent with squamous cell carcinoma (**Figures 1L–S**).

Subsequently, the patient underwent esophagoscopy, gastroscopy, and duodenoscope to rule out upper gastrointestinal tract origin tumors. Combined with previous bronchoscopy and pathological findings, the diagnosis of PSCCT was confirmed. Based on previous case reports (1, 8, 9) and retrospective studies (5, 6), the diagnosis was adequate. We also ran a genetic test to assess tumor mutation burden. Unfortunately, BRAF, HRAS, NRAS, TERT mutations and RET, PPARG, NTRK3 fusion were all negative. Due to the unresectable locally advanced tumor, the patient was treated with Anlotinib (Focus V^®^, Jiangsu Chia-Tai Tianqing Pharmaceutical and Advenchen Laboratories) 12 mg once daily taken orally continuously for 2 weeks of each 3-week cycle combined with Sintilimab (Tyvyt^®^, Innovent Biologics and Eli Lilly and Company) 200 mg once every 21 days. The patients were evaluated before every 3 treatment cycles. Surprisingly, after 2 courses, the volume of the tumor reduced significantly (maximum diameter from 11.17cm to 8.52cm, **Figure 1B**), and the symptoms of compression were relieved. Except for reversible leucopenia and skin pruritus, other laboratory tests were normal, and there were no other complications. MRI images showed almost no significant abnormalities in the original tumor site after 5 courses (**Figures 1C, E, G**).

On April 25, 2021, the patient was sent to the local hospital for malignant vomiting, anorexia, and disturbance of consciousness. He had an unexplained elevation of AST, ALT, GGT, and bilirubin with progressive aggravation (**Supplement Table 1**). He was diagnosed with fulminant liver failure and autoimmune liver disease in the local hospital. Three days later, the patient died there. The timeline of the major diagnosis and treatment is shown in **Supplement Table 2**.

## 3 Discussion

PSCCT usually appears in 50 to 60 years old women. Patients normally present with dyspnea or dysphagia for the airway and esophagus compressed by the tumor, and hoarseness for tumor invading recurrent laryngeal nerve (10). The origin of PSCCT is controversial because thyroid tissue itself does not have squamous cells. There are two common hypotheses, the “embryonic rest” theory (which holds that cancer cells develop from the remnants of the thyroglossal canal) and the “metaplasia” theory (4).

Pathologically, PSCCT showed islands of differentially differentiated squamous cells with distinct atypia and intercellular bridging (11). Immunohistochemistry is essential for the diagnosis of PSCCT. Typical positive stains include keratin, thyroglobulin, P40, P63, and Ki67 (1, 6, 12). P63 and P40 are sensitive markers of squamous cell differentiation, and P40 is more specific than P63 in recognizing squamous cell carcinoma (13). CK5/6 helps differentiate whether poorly differentiated squamous cell carcinoma is primary (14). Ki67 index is a marker of cell proliferation and about 80% PSCCT has a Ki67 index of 30% or above (6). Our patient Ki67 index was over 95%, demonstrating tumor proliferation and not just squamous metaplasia.

PSCCT has a poor response to radiotherapy, chemotherapy, and radioactive iodine ablation (5–7). Reviewing the literature on PSCCT in recent years (**Table 1**), surgery is a useful method for improving the survival rate. Moreover, it is worth noting that TKIs are increasingly being used as a trial treatment and are expected to prolong the survival of patients with PSCCT.

**Table 1:** Treatment and survival of primary squamous cell carcinoma of the thyroid.

Reference, year	No. of cases	Tumor	Survival	Treatment
Brandenburg et al., 2021 (15)	1	PSCCT (with BRAF V600E mutation)	>12 months	Dabrafenib and Trametinib
Xin et al., 2021 (16)	1	PSCCT	<1 months	Surgery
Sun et al., 2020 (17)	1	PSCCT	>23 months	Surgery+ RT
Torrez et al., 2020 (18)	1	PSCCT (with BRAF mutation and high PD-L1 expression)	>4 months	Surgery+ RT+ Dabrafenib and Trametinib
Wang et al., 2019 (10)	12	PSCCT	10.5 months	Surgery (6) + RT (11) +CT (6)
Raggio et al., 2019 (19)	1	PSCCT	<1 months	CT
De Cesare et al., 2019 (20)	1	PSCCT	10 months	Surgery+ CT
Yasumatsu et al., 2018 (21)	10	PSCCT	1-year survival rate (22.7%)	Surgery (8) + RT (9) + Lenvatinib

*CT, chemotherapy; RT, radiotherapy.

In the past decade, one significant advance in thyroid cancer is the molecular pathogenesis (22). The pathogenesis of thyroid cancer involves multiple steps. Changes in oncogenes and anti-oncogenes lead to abnormal cell proliferation, while changes in vascular growth factors lead to tumor invasion and spread (23). MAPK and PI3K/AKT are the most representative pathways which affect cell proliferation and differentiation (24). Common genetic changes in the MAPK pathway include proximal RET/PTC translocation and distal RAS and BRAF mutations, while common mutations in the PI3K pathway include RAS, PIK3CA, AKT1 and PTEN mutations (25). These two signaling pathways are coupled to the receptor tyrosine kinase (RTK), a transmembrane glycoprotein that conveys extracellular growth signals. RTK is the most frequently altered signaling pathway in all cancer types (26). Abnormal activation of RTK is related to the proliferation and metastasis of a variety of tumor cells (27). RTK inhibitors (TKIs) have shown significant antitumor activity in various tumors, including thyroid cancer (28, 29). Sorafenib, vandetanib and cabozantinib have been shown to be first-line therapies for advanced thyroid malignancies (30). Unfortunately, the classic genetic changes in MAPK and PI3K pathways were not detected in our patients, so there are no specific targeted drugs. Therefore, we chose to use Anlotinib, which has more targets and similar tolerances (31).

Anlotinib is a novel tyrosine kinase inhibitor that targets vascular endothelial growth factor receptor (VEGFR), fibroblasts growth factor receptor (FGFR), platelet-derived growth factor receptor (PDGFR) and C-Kit. The drug has a broad-spectrum inhibitory effect on cell growth and angiogenesis (32). Anlotinib is currently approved as a third-line drug in non-small cell lung cancer (NSCLC) (33), and has been studied in differentiated thyroid cancer and medullary thyroid cancer. In preclinical models of papillary thyroid carcinoma (PTC) and anaplastic thyroid carcinoma (ATC), Ruan et al. (34) found that Anlotinib can affect cell viability by interfering with spindle assembly, leading to G2/M phase stagnation and activation of TP53. In addition, Anlotinib inhibits the migration of tumor cells *in vitro* and affects the growth of xenograft thyroid tumors *in vivo*. In a randomized, placebo-controlled Phase IIB trial, 91 patients with histopathological proven and unresectable medullary thyroid carcinoma (MTC) were enrolled. Anlotinib significantly extended median progression-free survival (11.1 to 20.7 months) (35). These studies demonstrate the therapeutic potential of Anlotinib in thyroid tumors.

Immune checkpoint proteins, including programmed cell death receptor 1 (PD-1), are involved in immune regulation of tumor (36). They may be utilized by tumor cells expressing PD-L1 to evade immune surveillance (37). Inhibiting immune checkpoints can promote antitumor immunity, thereby eliminating tumor cells (38). PD-1/PD-L1 inhibitors have been widely used in tumor immunotherapy, including thyroid tumors (39–41). Some subtypes, such as ATC, show a high level of PD-L1 expression, suggesting that such patients may be sensitive to PD-1/PD-L1 inhibitors (42). Currently, more than a dozen clinical studies have used PD-1/PD-L1 inhibitors in the treatment of unresectable, recurrent, and/or metastatic thyroid tumors (39), demonstrating that PD-1/PD-L1 inhibitors may be a “life-saving” option for advanced thyroid tumors.

Sintilimab is a monoclonal antibody of human origin IgG4. It binds to PD-1, blocking the interaction between PD-1 and its ligand, thereby restoring the function of endogenous T cell (43). According to a multicenter Phase II study, average PD-1 receptor occupancy ≥95% (44). Sintilimab was first used for relapsed or refractory Hodgkin’s lymphoma (43). Although it was widely used in various solid tumors such as NSCLC and squamous cell esophageal carcinoma, showing exciting antitumor activity (45), it has not been studied in thyroid tumors. We examined PD-L1 expression in accordance with previous clinical studies of Sintilimab (46). The 22C3 pharmDx (Dako) kit was used for staining, and positive and negative controls were set on the same slice. The whole experiment was carried out under the standard procedure (47). We used the combined positive score (CPS) for assessment because CPS may be more relevant to the benefit of anti-PD-1 therapy than the tumor proportion score (48). The PD-L1 CPS≥10 in thyroid tumor tissue sections indicated that the patient was likely to benefit from the treatment of Sintilimab (**Supplement Figure 1**).

**Figure 2:**
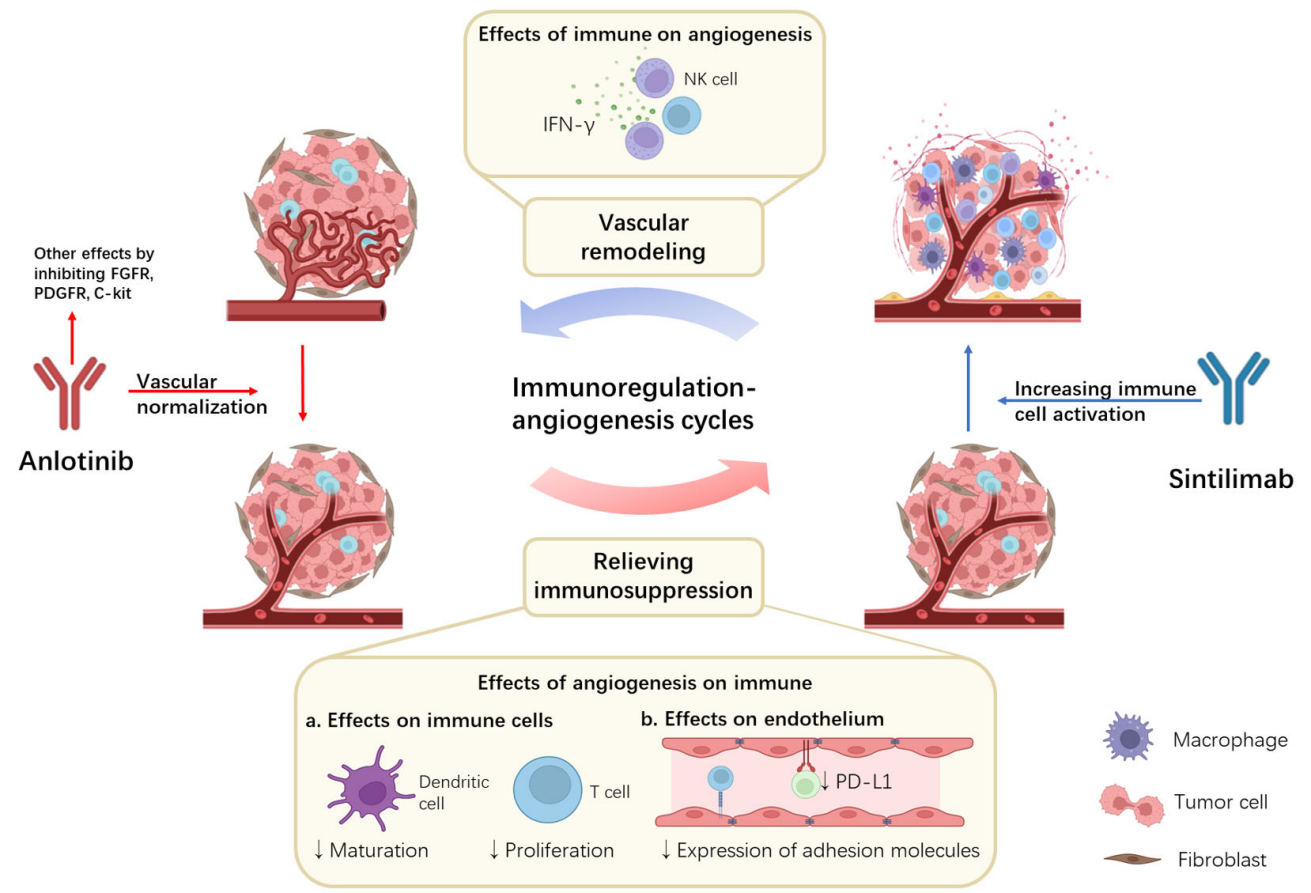
Potential synergistic mechanisms of Anlotinib combined with Sintilimab and the immunoregulation-angiogenesis cycles. Anlotinib inhibits angiogenesis by targeting VEGF. At the same time, the inhibition of VEGF can also relieve immunosuppression in the tumor microenvironment by directly affecting immune cells and indirectly affecting endothelium. Sintilimab binds to PD-1 and blocks its binding to PD-L1 and PD-L2, activating immune cells. At the same time, activated immune cells release large amounts of IFN-γ, further leading to vascular remodeling. Positive feedback is formed between immune regulation and angiogenesis.

A growing amount of evidence describes the interaction between tumor immune microenvironment and angiogenesis, which has also been mentioned in previous reviews (49–51). In short, VEGF not only promotes angiogenesis but also affects the immune regulation, helping tumor cells evade immune surveillance (52). It is currently believed that VEGF attenuates the antitumor response through two modes of action. Firstly, VEGF can directly affect the immune cells, for example, inhibiting T cell differentiation and dendritic cell maturation (53, 54). Secondly, by affecting the adhesion molecules of endothelial cells, VEGF affects the transport of lymphocytes and T cells to tumor cells (55). As for Anlotinib, a previous study found that it can down-regulate the expression of PD-L1 on vascular endothelial cells, thereby increasing CD8^+^T cell infiltration, decreasing FoxP3^+^T cell aggregation, and relieving immunosuppression (56). Conversely, the enhanced immune microenvironment also affects angiogenesis. IFN-γ is thought to play an essential role in this process. Previous studies have shown that IFN-γ secreted by activated CD8+T cells can mediate anti-angiogenesis (57). IFN-γ has also been shown to directly down-regulate delta-like protein 4 and VEGF mRNA expression on endothelial cells (58, 59). Therefore, we believed that there was a positive feedback regulation before immune regulation and angiogenesis. Accordingly, we speculated the mechanism by which the coordination of Anlotinib and Sintilimab plays an antitumor role (**Figure 2**).

Clinical studies of multiple solid tumors have also reported clinical benefits from the combination of ICIs and antiangiogenic agents (60–64). Among them, the combination of Anlotinib and PD-1 inhibitors (including Sintilimab) has also received widespread attention (65, 66). (**Table 2**) Moreover, because Anlotinib has a broader range of targets than VEGF inhibitors, it is also thought to have better efficacy (66).

**Table 2:** Recent clinical studies involving the combined use of ICIs and antiangiogenic agents.

Year, ref	Identifier	Phase	Drugs	Indication	Effects	AEs
2021 (66)	NCT03628521	IB	Anlotinib+ Sintilimab	NSCLC	ORR=72.7%, media PFS=15 months	Grade 3 or higher TRAE (54.5%)
2021 (65)	/	/	Anlotinib+ PD-1 inhibitor	SCLC/NSCLC	SCLC, PFS=8 months; NSCLC, PFS=8 months	No new or unexpected AEs
2020 (64)	NCT02501096	IB/II	Lenvatinib+ Pembrolizumab	RCC/endometrial cancer/melanoma/SCCHN/NSCLC/urothelial cancer	ORR_wk24_=RCC (63%)/endometrial cancer (52%)/melanoma (48%)/SCCHN (36%)/NSCLC (33%)/urothelial cancer (25%)	Fatigue (58%), diarrhea (52%), hypertension (47%), and hypothyroidism (42%).
2020 (63)	NCT02501096	IB/II	Lenvatinib+ Pembrolizumab	Advanced endometrial cancer	ORR_wk24 =_ 38.0%, media PFS=7.4 months	Grade 3 or higher TRAE (66.9%)
2019 (62)	NCT02443324	IA/B	Ramucirumab+ Pembrolizumab	NSCLC, gastro-esophageal cancer, urothelial carcinomas	median follow-up= 32.8 months	Grade 3 or higher TRAE (24%)
2019 (60)	NCT02853331	III	Axitinib+ Pembrolizumab/Sunitinib	RCC	ORR (59.3%: 35.7%), media PFS (15.1: 11.1 months)	Grade 3 or higher TEAE (75.8%: 70.6%)
2018 (61)	IMmotion150	II	Atezolizumab/Atezolizumab+ Bevacizumab/Sunitinib	RCC	media PFS (6.1: 11.7: 8.4 months), ORR (25%: 32%: 29%)	Grade 3 or higher TEAE (40%: 63%: 69%)

*NSCLC, non-small cell lung cancer; SCLC, small cell lung cancer; RCC, renal cell carcinoma; SCCHN, squamous cell carcinoma of the head and neck; ORR, objective response rate; PFS, progression-free survival; AE, adverse events; TRAE, treatment-related adverse events; TEAE, treatment-emergent adverse events.

Therefore, our patient received Anlotinib and Sintilimab combination therapy. The result was outstanding. After 2 courses, the tumor significantly decreased, and the compression symptoms were relieved. The tumor was almost invisible after five months of treatment, indicating the ability of ICIs and TKIs combination therapy.

However, the increasing adverse events (AEs) associated with combination therapy should be considered. Although the combination therapy in the current study was generally well tolerated (60–64), the incidence of treatment-related hepatic AE was slightly higher than in the whole study population (although elevated liver enzymes were generally Grade 1/2) (65). Immune-related adverse events (IRAE) caused by immunotherapy have also been widely concerned (67). This patient died of fulminant liver failure and autoimmune liver disease after 5 months of treatment. Typically, most IRAEs occur 3-6 months after ICIs administration (68) and are sensitive to steroids, which resolve within 6-12 weeks (69). About 5% of patients developed immune-associated hepatitis, presenting with an unexplained elevation of AST or ALT (70). Most patients lack symptomatic but abnormal in laboratory tests (71). In addition, Anlotinib by itself causes hypertriglyceridemia and hypercholesterolemia (32), which in combination may increase the risk of immune liver disease. Therefore, the AEs of combination therapy, especially immune liver disease, need special attention.

## 4 Conclusion

In summary, PSCCT is a rare disease characterized by locally advanced symptoms and poor prognosis. The treatment of PSCCT is limited, and surgical resection is currently the dominant treatment. TKIs combined with ICIs maybe is an effective way for PSCCT treatment. Since this study is a single case report, more randomized controlled clinical studies are expected to be carried out in the future to determine the efficacy. In addition, immune-related adverse reactions should be careful. When TKIs plus ICIs are used together, we need to pay attention to the occurrence of severe liver failure.

## Data availability statement

The original contributions presented in the study are included in the article/**Supplementary Material**. Further inquiries can be directed to the corresponding authors.

## Ethics statement

The studies involving human participants were reviewed and approved by the Ethics Committee of Shanghai Ninth People’s Hospital, Shanghai JiaoTong University School of Medicine (SH9H-2020-T346-1). The patients/participants provided their written informed consent to participate in this study. Written informed consent was obtained from the patients/participants for the publication of this case report.

## Author contributions

ZL: literature research and manuscript preparation. MY: manuscript preparation. FZ: treatment of the patient and clinical data collection. CZ: treatment of the patient and guarantee of the integrity of the whole research process. All authors contributed to the article and approved the submitted version.
