# Supplementary material for: Anlotinib combined with Sintilimab is win-win cooperation for primary squamous cell carcinoma of the thyroid: A case report and literature review
Source: Front Oncol. 2023 Mar 16;13:976415. doi: 10.3389/fonc.2023.976415 (PMC10062477; doi:10.3389/fonc.2023.976415)
Supplement: Supplementary file 1 [file DataSheet_1.docx]

Supplementary Material

**Supplementary Table 1.** Results of laboratory examination on April 25, 2021.

| **Inspection items** | **Inspection Results** | **Reference range** |
| --- | --- | --- |
| Total bilirubin | 251.1 μmol/L | 5.0-21.0 μmol/L |
| Conjugated bilirubin | 115.2 μmol/L | 0-5.0 μmol/L |
| Unconjugated bilirubin | 80.8 μmol/L | 0-19.0 μmol/L |
| Albumin | 27.8 g/L | 35.0-50.0 g/L |
| Globulin | 40.9 g/L | 20.0-35.0 g/L |
| the ratio of albumin to globulin, AG | 0.68 | 1.20-2.50 |
| Glutamic pyruvic transaminase, ALT | 1367 U/L | 21-72 U/L |
| Glutamic oxaloacetic transaminase, AST | 842 U/L | 15-46 U/L |
| γ-glutamyl transpeptidase, GGT | 225 U/L | 15-73 U/L |
| Alkaline phosphatase, ALP | 252 U/L | 38-126 U/L |

**Supplementary Table 2.** Timeline of important historical and current information.

| **Event** | **Date** |
| --- | --- |
| **Historical information** |  |
| Diagnosed as thyroid tumor in the First People's Hospital of Suqian | July 14, 2020 |
| A tracheal stent was placed in Shanghai Chest Hospital | November 7, 2020 |
| **Current information** |  |
| Hospitalization in our hospital | November 19, 2020 |
| Ultrasound, MRI, and other basic examinations | November 19, 2020 |
| Right neck lymph node biopsy and right partial thyroidectomy | November 26, 2020 |
| Genetic testing and endoscopy | December 2020 |
| Treatment and follow-up | from December 2020 to April 2021 |

**Supplementary Figure 1.**
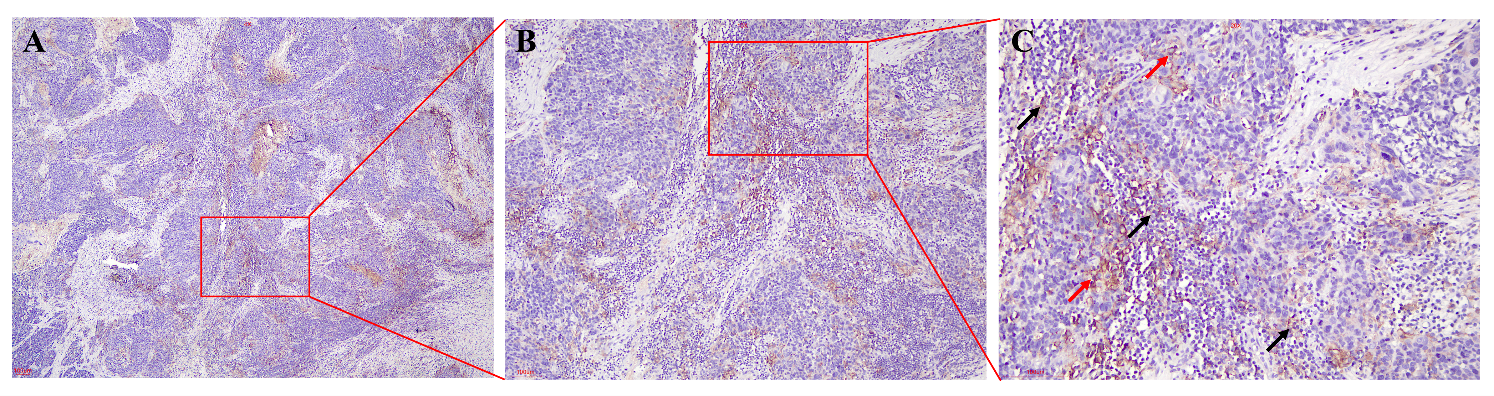


**Supplementary Figure 1**. PD-L1 stain of PSCCT. **(B)** and **(C)** are local enlarged images of **(A)** and **(B)**, respectively. Deep staining of cell membrane represents PD-L1 positive. The red arrow in **(C)** marks PD-L1 positive tumor cells, and the black arrow marks PD-L1 positive immune cells. (A×40, B×100, C×200)
